# Supplementary material for: Flavones from Combretum quadrangulare Growing in Vietnam and Their Alpha-Glucosidase Inhibitory Activity
Source: Molecules. 2021 Apr 26;26(9):2531. doi: 10.3390/molecules26092531 (PMC8123651; doi:10.3390/molecules26092531)
Supplement: Supplementary file 1 [file molecules-26-02531-s001.zip › molecules-1198762-supplementary.pdf]

FLAVONES FROM *COMBRETUM QUADRANGULARE* GROWING IN VIETNAM AND THEIR ALPHA-GLUCOSIDASE INHIBITORY ACTIVITIVES

Thi-Bich-Ngoc Dao[1], Truong-Minh-Tri Nguyen[1], Thi-Minh-DinhTran[2], Nguyen-Minh-An Tran[3], Chuong Hoang Nguyen[4], Van-Quy Nguyen[1], Thi-Hoai-Thu Nguyen[5], Huu-Hung Nguyen[6], Jirapast Sichaem[7], Cong-Luan Tran[8*], Thuc-Huy Duong[1*]

1 Department of Chemistry, University of Education, 280 An Duong Vuong Street, District 5, Ho Chi Minh City 72711, Vietnam; ngocdaosph@gmail.com (T.-B.-N.D.); nguyentruongminhtri99@gmail.com (T.-M.-T.N.); nguyenvanquysphoa@gmail.com (V.-Q.N.)
2 Department of Biology, Ho Chi Minh City University of Education, 280 An Duong Vuong Street, District 5, Ho Chi Minh City 72711, Vietnam; dinhttm@hcmue.edu.vn
3 Industrial University of Ho Chi Minh City, Ho Chi Minh City 71420, Vietnam; trannguyenminhan@iuh.edu.vn
4 University of Science, Vietnam National University, Ho Chi Minh City 72711, Vietnam; nhchuong@hcmus.edu.vn
5 Faculty of Basic Sciences, University of Medicine and Pharmacy at Ho Chi Minh City, 217 Hong Bang Street, District 5, Ho Chi Minh City 72714, Vietnam; nguyenthihoaithu@ump.edu.vn
6 Faculty of Technology, Van Lang University, 45 Nguyen Khac Nhu, District 1, Ho Chi Minh City 71013, Vietnam; hung.nh@vlu.edu.vn
7 Research Unit in Natural Products Chemistry and Bioactivities, Faculty of Science and Technology, Thammasat University Lampang Campus, Lampang 52190, Thailand; jirapast@tu.ac.th
8 Faculty of Pharmacy and Nursery, Tay Do University, Can Tho 94000, Vietnam
* Correspondence: tcluan@tdu.edu.vn (C.-L.T.); huydt@hcmue.edu.vn (T.-H.D.); Tel.: +84-903855528 (C.-L.T.); +84-919011884 (T.-H.D.)

Abstract: *Combretum quadrangulare* Kurz is widely used in folk medicine in Eastern Asia and is associated with various ethnopharmacological properties including hepatoprotective, antipyretic, analgesic, antidysenteric, and anthelmintic activities. Previous phytochemical investigations reported the presence of numerous triterpenes (mostly cycloartanes, ursanes, lupanes, and oleananes) along with dozen of flavonoids [1-7]. However, the extracts of *C. quadrangulare* and isolated flavonoids have not been evaluated their alpha-glucosidase inhibition. In the frame of our efforts dedicated to the chemical investigation of Vietnamese medicinal plants and their biological activities, a phytochemical study of the MeOH extract of the leaves of *C. quadrangulare* using bioactive guided isolation was undertaken. In this paper, the isolation and structure elucidation of twelve known compounds, 5-hydroxy-3,7,4′-trimethoxyflavone (**1**), ayanin (**2**), kumatakenin (**3**), rhamnocitrin (**4**), ombuin (**5**), myricetin-3,7,3',5'-tetramethyl ether (**6**), gardenin D (**7**), luteolin (**12**), apigenin (**13**), mearnsetin (**14**), isoorientin (**15**) and vitexin (**16**) were reported. Bromination was applied to compounds **2** and **3** to provide four new synthetic analogues **8-11**. All isolated and synthesized compounds were evaluated for alpha-glucosidase inhibition and antibacterial activity. Compounds **4** and **5** showed moderate antibacterial activity against methicillin-resistant *Staphylococcus aureus* while others are inactive. All compounds failed to reveal any activity toward extended spectrum beta-lactamase-producing *Escherichia coli*. Compounds **2**, **4**, **6-9**, and **11-14** showed good alpha-glucosidase inhibition with IC_{50} values in the range of 30.5-282.0 μM. The kinetic of enzyme inhibition showed that **8** and **11** were noncompetitive type inhibition against alpha-glucosidase. *In silico* molecular docking model indicated that compounds **8** and **11** were potential inhibitors against enzyme *α*-glucosidase.

Keywords: *Combretum quadrangulare* Kurz, flavonoid, alpha-glucosidase, antibacterial, molecular docking

Table S1. ^{1}H NMR (500 MHz) data of isolated flavonones; (Acetone- d_6, *DMSO-d_6, δ in ppm).

Position	2<br>δ_H mult. (*J*)	3*<br>δ_H mult. (*J*)	4<br>δ_H mult. (*J*)	5<br>δ_H mult. (*J*)	6<br>δ_H mult. (*J*)	7<br>δ_H mult. (*J*)	14<br>δ_H mult. (*J*)	15*<br>δ_H mult. (*J*)	16*<br>δ_H mult. (*J*)
3								6.77 s	6.64 s
6	6.31 d 2.0	6.37 d 2.0	6.26 d 2.0	6.26 d 2.0	6.32 d 2.0		6.27 d 1.5	6.27 s	
7									
8	6.66 d 2.0	6.74 d 2.5	6.50 d 2.0	6.51 d 2.0	6.68 d 2.0		6.52 d 2.0		6.46 s
9									
10									
1′									
2′	7.79 d 2.0	7.98 d 9.0	8.03 d 8.5	7.78 d 2.0	7.54 s	7.57 d 2.0	7.38 s	8.01 d 8.0	7.39 d 2.0
3′		6.95 d 9.0	7.02 d 8.5					6.89 d 8.0	
4′									
5′	7.01 d 8.0	6.95 d 9.0	7.02 d 8.5	7.01 d 8.5		6.97 d 8.5		6.89 d 8.0	6.87 d 8.0
6′	7.70 dd 8.5, 2.5	7.98 d 9.0	8.03 d 8.5	7.68 dd 8.5, 2.0	7.54 s	7.59 dd 8.5, 2.0	7.38 s	8.01 d 8.0	7.40 dd 8.0, 2.0
1′′								4.68 d 10.0	4.58 d 10.0
2′′								3.84 t-like 9.5	4.04 t-like 9.5
3′′								3.24-3.37 m	3.11-3.22 m
4′′									
5′′									
6′′								3.75 brd 10.5<br>3.53 dd 11.5, 5.5	3.67 brd 10.5<br>3.40 overlap
3-OCH$_3$	3.90 s	3.80 s			3.92 s				
4-OCH$_3$									
6-OCH$_3$						4.11 s			
7-OCH$_3$	3.94 s	3.86 s	3.87 s	3.95 s	3.91 s	3.99 s			
8-OCH$_3$						3.98 s			
3′-OCH$_3$					3.93 s				
4′-OCH$_3$	3.91 s			3.89 s		3.92 s			
5′-OCH$_3$					3.93 s				
4′-OH					8.10 s				
5-OH			12.80 s	12.79 s	12.75 s		12.08, s	13.16 s	13.55 s
3′-OH							8.45, brs		
5′-OH							8.45, brs		

Table S2. ^{13}C- NMR (125 MHz) data of isolated flavonoids; (Acetone- *d6*, *DMSO-*d6*, δ in ppm)

Position	2<br>δ_C mult.	3<br>δ_C mult.	4<br>δ_C mult.	5<br>δ_C mult.	6<br>δ_C mult.	7<br>δ_C mult.	14<br>δ_C mult.	15*<br>δ_C mult.	16*<br>δ_C mult.
2	156.9	156.4	147.3	148.4	156.9	166.4	145.3	163.9	163.6
3	139.5	137.9	136.6	139.4	140.4	104.0	137.3	102.4	102.7
4	179.6	178.1	176.0	179.6	179.7	184.7	175.9	182.1	181.8
5	162.9	161.0	161.0	163.3	163.0	147.2	161.3	160.4	160.7
6	98.5	97.8	99.5	99.5	98.6	137.7	98.4	98.2	108.9
7	166.6	165.2	165.0	165.0	166.7	154.4	164.5	162.7	162.8
8	92.9	92.4	94.6	94.6	93.1	133.0	93.5	104.6	93.6
9	157.7	156.0	158.0	157.9	157.8	144.6	156.8	156.0	156.2
10	106.6	105.2	104.7	105.99	106.6	107.9	103.4	104.0	103.2
1′	123.4	120.5	122.8	122.9	121.6	123.5	125.5	121.6	121.3
2′	116.1	130.2	131.3	116.2	107.5	116.9	107.4	128.9	113.2
3′	148.3	115.7	116.5	156.8	148.8	149.6	150.5	115.8	145.8
4′	150.6	160.3	159.4	150.6	139.8	152.5	137.4	161.1	149.9
5′	112.7	115.7	116.5	112.8	148.8	110.6	150.5	115.8	116.0
6′	122.8	130.2	131.3	123.4	107.5	121.9	107.4	128.9	119.0
1′′								73.4	73.1
2′′								70.8	70.2
3′′								78.7	79.0
4′′								70.6	70.6
5′′								81.8	81.5
6′′								61.3	61.5
3-OCH_3	60.2	59.8			60.4		60.7		
4-OCH_3									
6-OCH_3						62.3			
7-OCH_3	56.4	56.1	60.3	56.5	56.5	61.5			
8-OCH_3						62.6			
3′-OCH_3					57.0				
4′-OCH_3	56.4			60.3		56.6			
5′-OCH_3					57.0				

Table S3. NMR comparison of **2** and **8**

	**8** (Acetone – *d*6)		**2** Acetone – *d*6)		**Ayanin** (Acetone – *d*6)	
	δH (ppm), J (Hz)	δC (ppm)	δH (ppm), J (Hz)	δC (ppm)	δH (ppm), J (Hz)	δC (ppm)
2		156.6		156.9		155.5
3		139.7		139.5		138.1
4		178.8		179.6		178.0
5		160.6		162.9		160.8
6		94.0	6.32 (d, J= 2.0 Hz)	98.5	6.38 (d, J= 2.1 Hz)	97.7
7		172.6		166.6		165.1
8		88.7	6.68 (d, J= 2.5 Hz)	92.9	6.73 (d, J= 2.1 Hz)	92.2
9		159.1		157.7		156.2
10		109.8		106.6		105.1
1'		122.1		123.4		122.1
2'	7.87 (d, J= 1.5 Hz)	110.9	7.78 (d, J= 2.5 Hz)	116.1	7.58 (d, J= 2.2 Hz)	115.0
3'		149.5		148.3		146.3
4'		152.3		150.6		150.2
5'		97.0	7.00 (d, J= 8.5 Hz)	112.7	7.12 (d, J= 9.5 Hz)	111.8
6'	8.18 (d, 1H, J= 2.0 Hz)	127.3	7.71 (dd, J= 8.5; 2.0 Hz)	122.8	7.59 (dd, J= 9.5, 2.2 Hz)	120.3
3-OCH_3	3.79 (s)	60.2	3.90 (s)	60.3	3.81 (s)	59.6
7-OCH_3	3.99 (s)	61.6	3.92 (s)	56.4	3.87 (s)	56.0
4'-OCH_3	3.97 (s)	56.6	3.95 (s)	56.4	3.87 (s)	55.6
5-OH	15.72 (s)		12.75 (s)		12.65 (s)	
3'-OH			8.5 (s)		9.5 (s)	

Table S4. NMR comparison of **3**, **9-11**

No.	**(9)** (DMSO-d_6)		**(10)** (DMSO- d_6)		**(11)** (DMSO- d_6)		**(3)** (DMSO- d_6)		**Kamatakenin** (DMSO- d_6)	
	δ_H (ppm), J (Hz)	δ_C (ppm)	δ_H (ppm), J (Hz)	δ_C (ppm)	δ_H (ppm), J (Hz)	δ_C (ppm)	δ_H (ppm), J (Hz)	δ_C (ppm)	δ_H (ppm), J (Hz)	δ_C (ppm)
2		155.8		155.3		156.9		156.5		156.4
3		137.8		137.9		138.9		137.9		138.3
4		178.0		177.6		177.7		178.6		178.5
5		160.8		157.0		161.6		161.5		161.4
6	6.69 (s)	96.1		92.7		99.7	6.37 (d, J= 2.0 Hz)	98.3	6.39 (d, J= 1.6 Hz)	98.2
7		161.3		160.8		166.2		165.5		165.6
8		87.0	7.03 (s)	91.9		95.0	6.74 (d, J= 2.5 Hz)	92.9	6.76 (d, J= 1.6 Hz)	92.8
9		151.7		156.3		159.2		156.9		156.8
10		105.5		105.6		108.7		105.7		105.7
1’		120.4		120.3		121.2		121.0		120.9
2’	8.07 (d, J= 9.0 Hz)	130.3	8.02 (d, J= 8.5 Hz)	130.3	8.28 (s)	132.2	7.98 (d, J= 9.0 Hz)	130.8	7.99 (d, J= 8.9 Hz)	130.7
3’	6.98 (d, J= 9.0 Hz)	115.7	6.96 (d, J= 9.0 Hz)	115.7		112.2	6.95 (d, J= 9.0 Hz)	116.2	6.97 (d, J= 8.6 Hz)	116.1
4’		160.6		160.5		161.2		160.8		160.8
5’	6.98 (d, J= 9.0 Hz)	115.7	6.96 (d, J= 9.0 Hz)	115.7		112.2	6.95 (d, J= 9.0 Hz)	116.2	6.97 (d, J= 8.6 Hz)	116.1
6’	8.07 (d, J= 9.0 Hz)	130.3	8.02 (d, J= 8.5 Hz)	130.3	8.28 (s)	132.2	7.98 (d, J= 9.0 Hz)	130.8	7.99 (d, J= 8.9 Hz)	130.7
3- OCH_3	3.83 (s)	59.7	3.82 (s)	59.7	3.87 (s)	61.1	3.80 (s)	60.3	3.87 (s)	60.2
7- OCH_3	3.97 (s)	57.3	3.98 (s)	57.3	3.92 (s)	59.9	3.86 (s)	56.6	3.81 (s)	56.6
4’-OH	10.39 (s)		10.34 (s)				10.31 (s)			
5-OH	12.85 (s)		13.47 (s)		13.41 (s)		12.66 (s)			

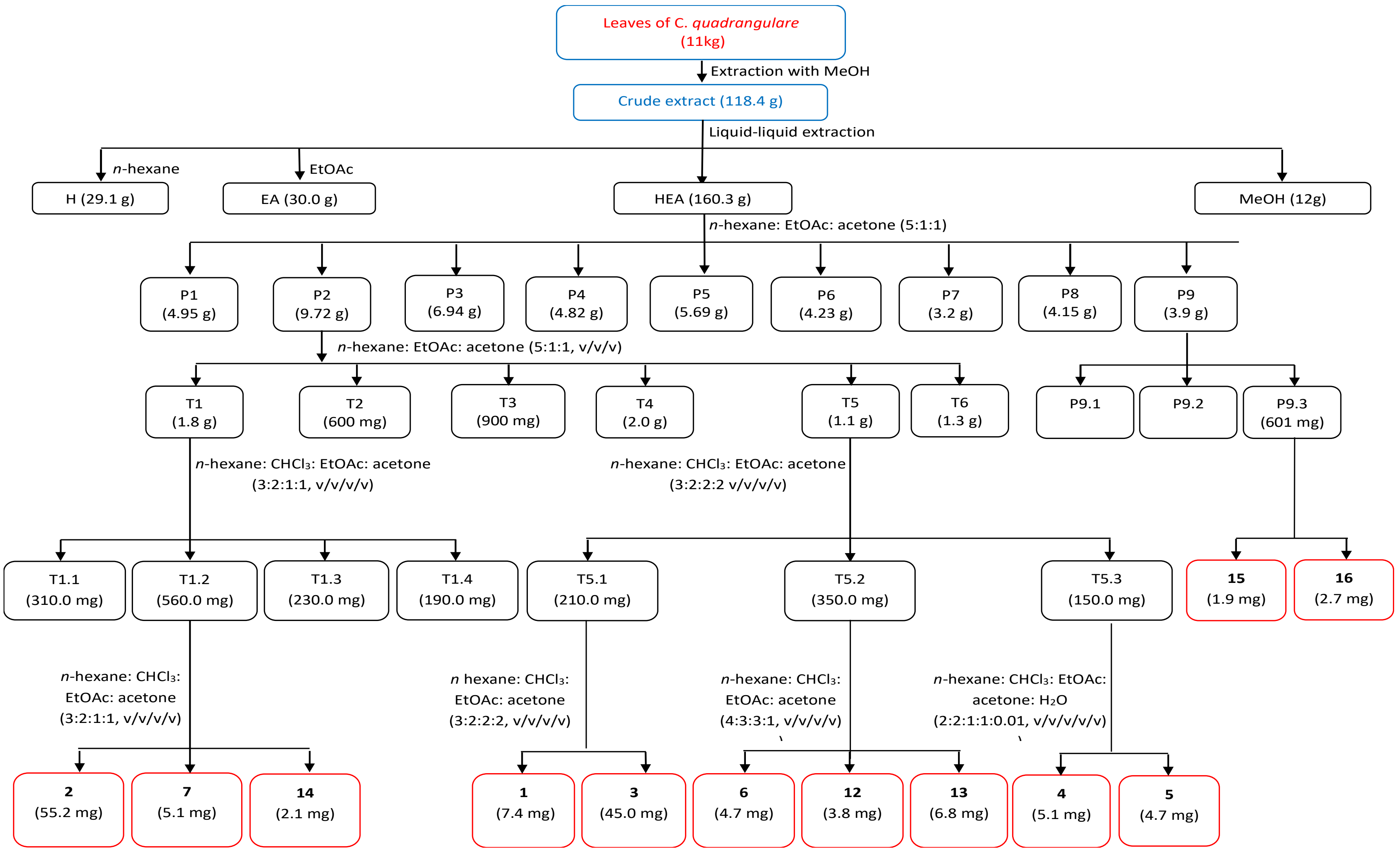

Figure S1. Isolation scheme of compounds from *Combretum quadrangulare* Kurz.

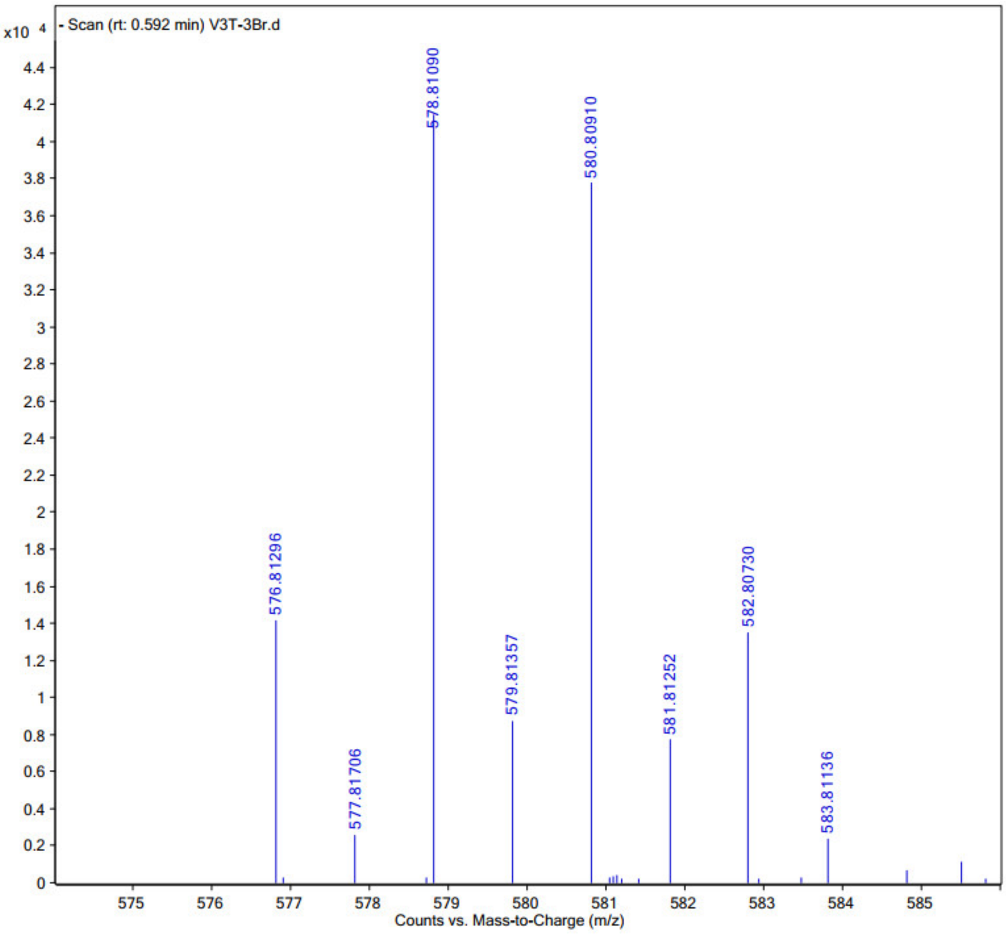

Figure S2. The HRESIMS spectrum of **8**

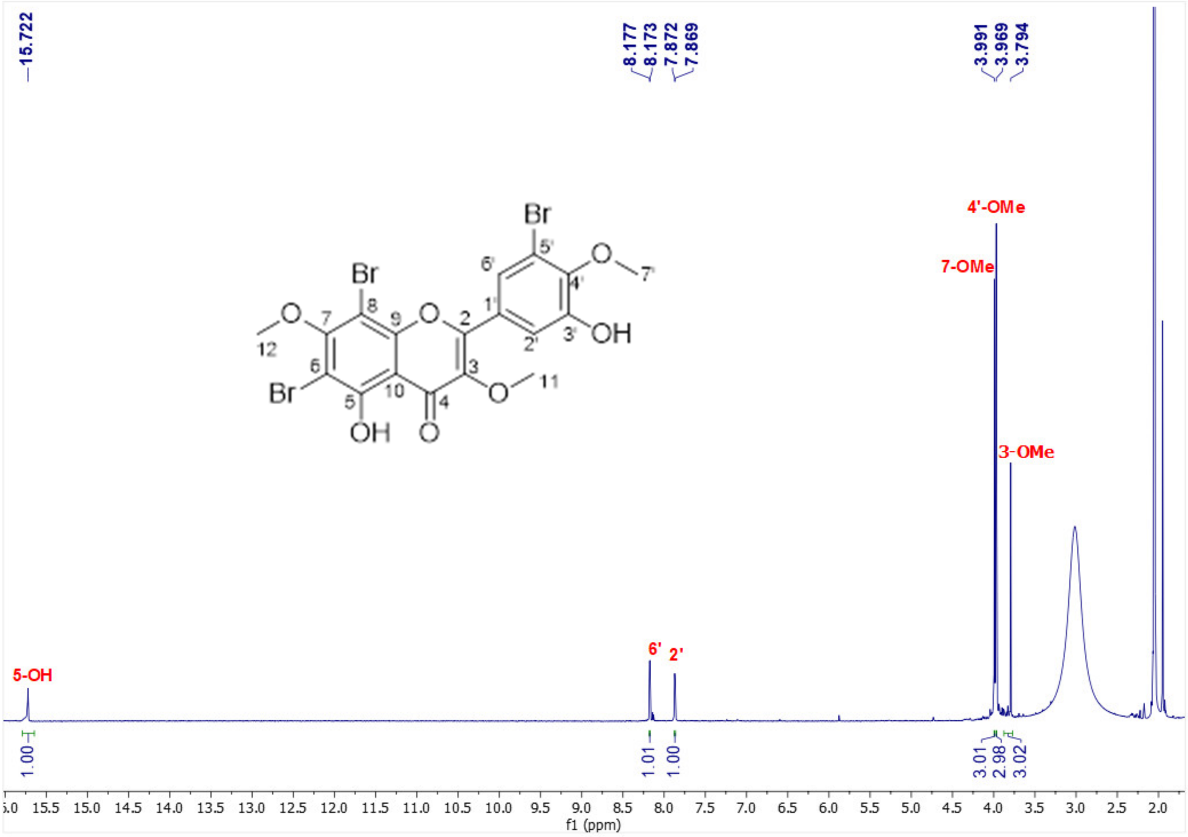

Figure S3. The ^{1}H NMR spectrum of **8** in Acetone--d_6

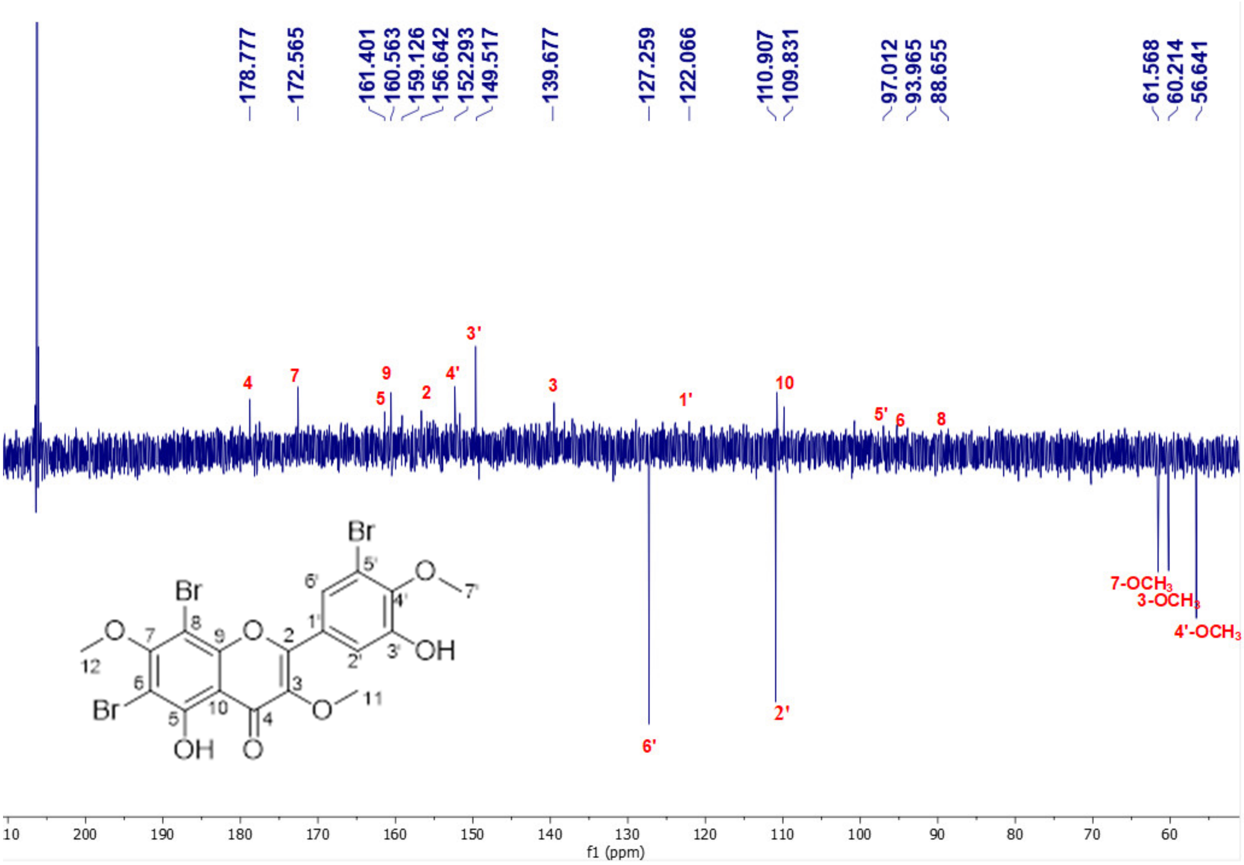

Figure S4. The ^{13}C NMR spectrum of **8** in Acetone--d_6

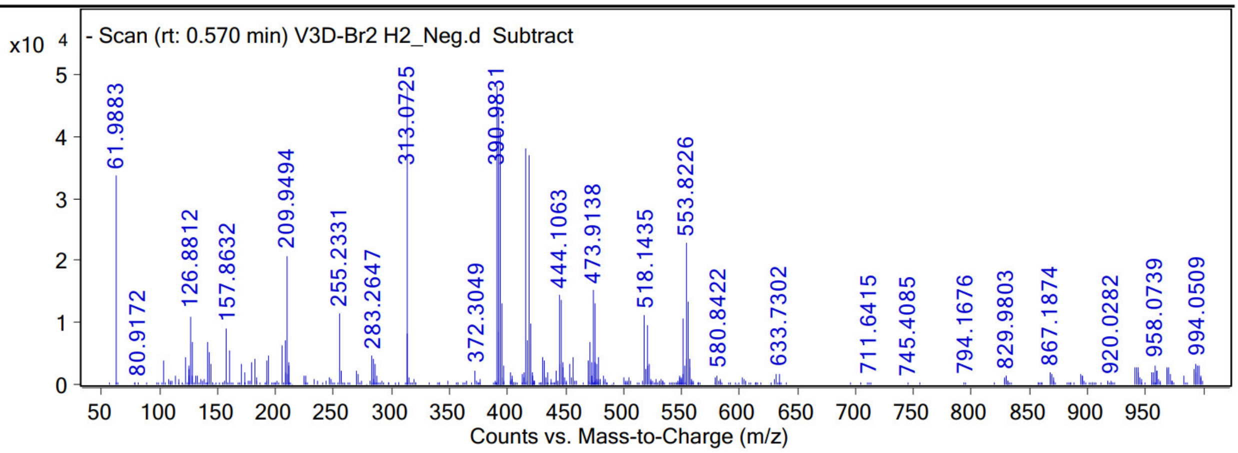

Figure S5. The HRESIMS spectrum of **TBV3D-Br2.1**

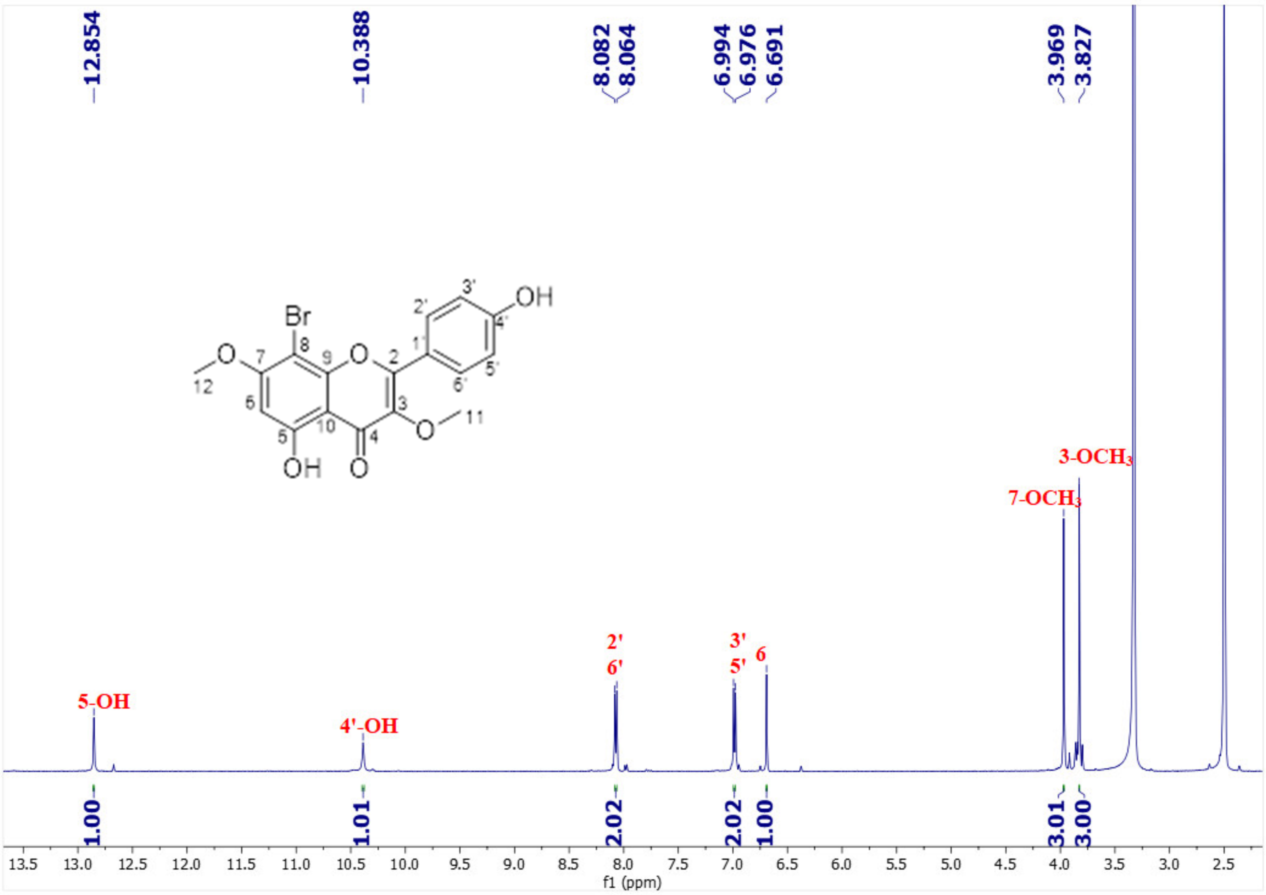

Figure S6. The ^{1}H NMR spectrum of **9** in DMSO--d_6

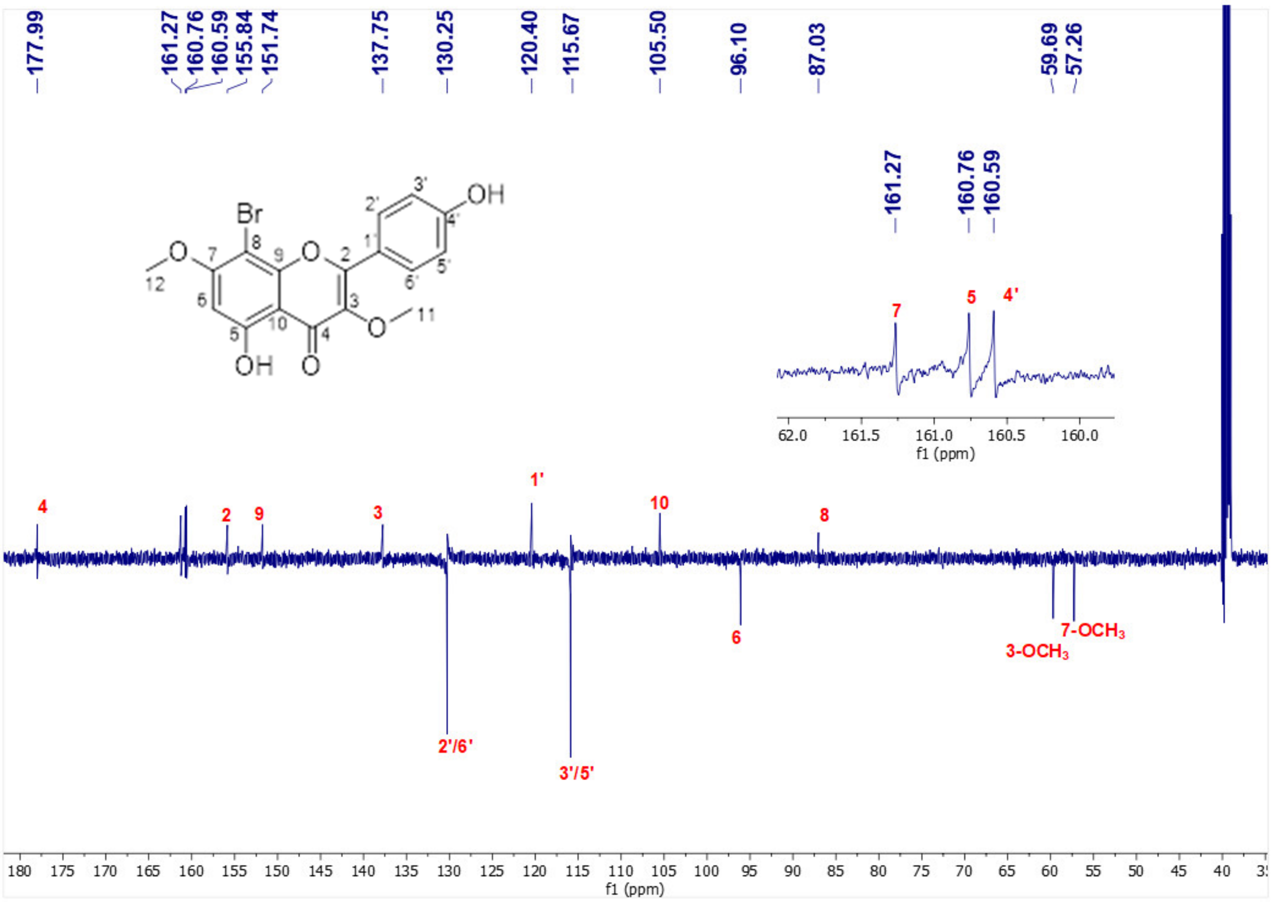

Figure S7. The ^{13}C NMR spectrum of **9** in DMSO--d_6

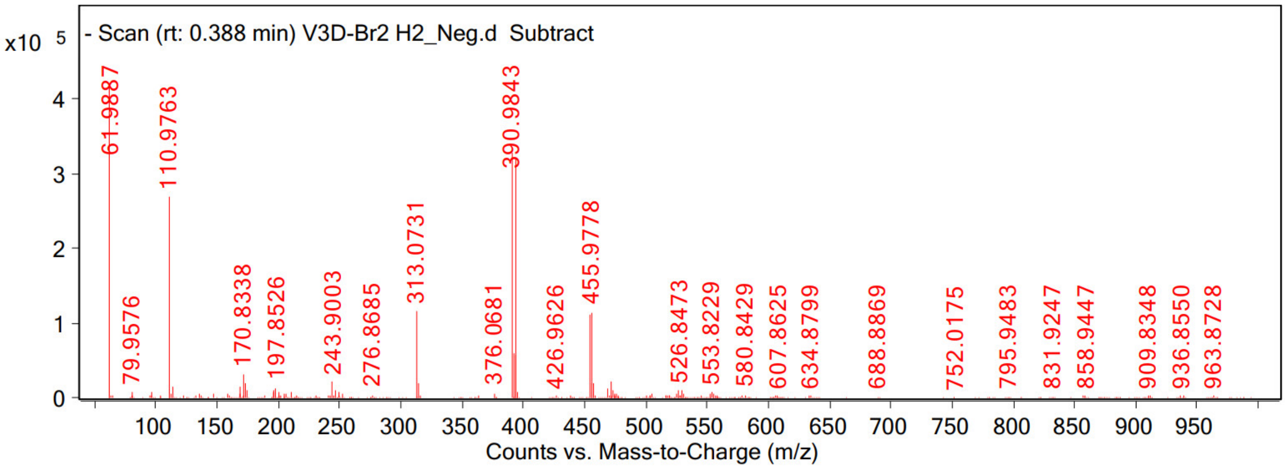

Figure S8. The HRESIMS spectrum of **10**

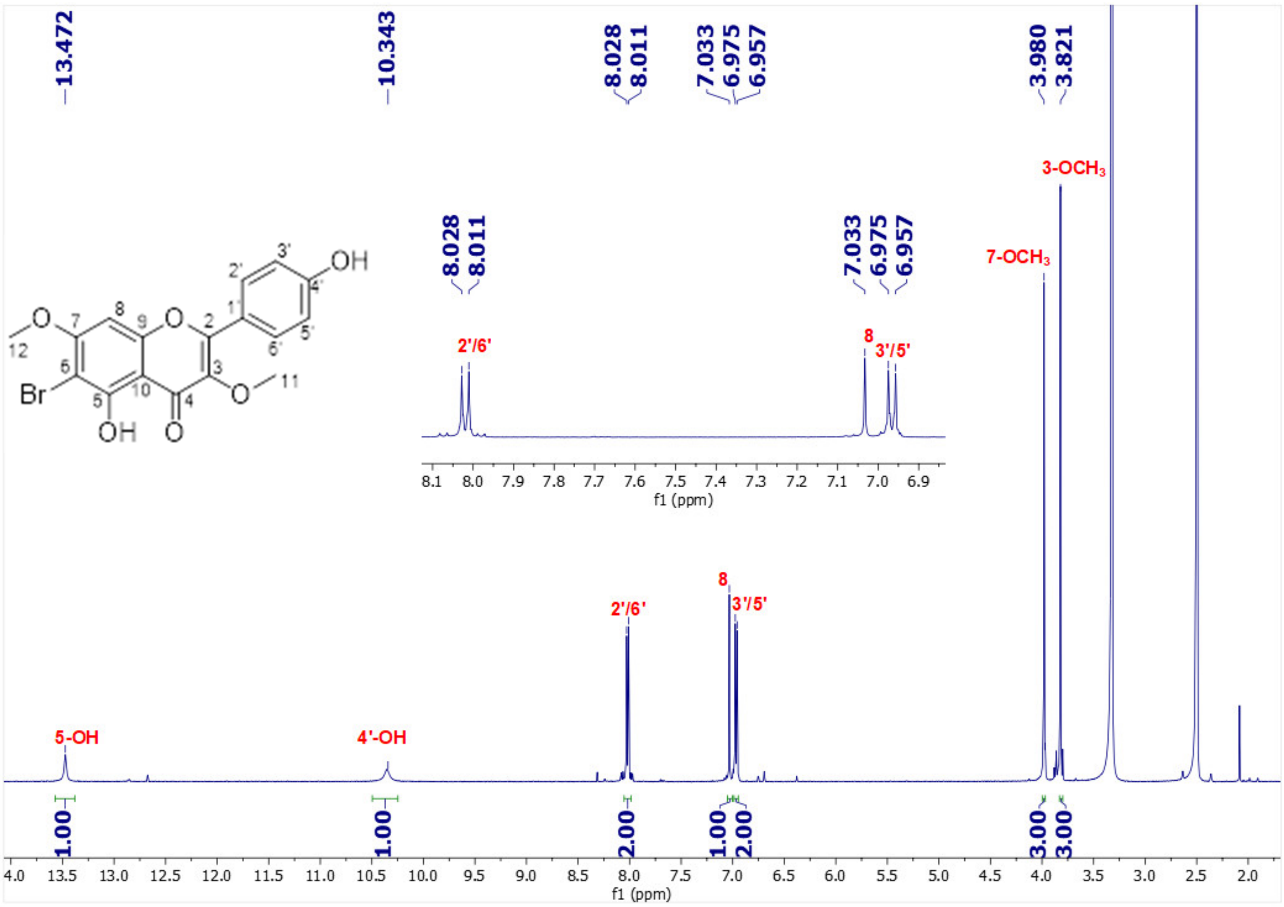

Figure S9. The ^{1}H NMR spectrum of **10** in DMSO--*d*6

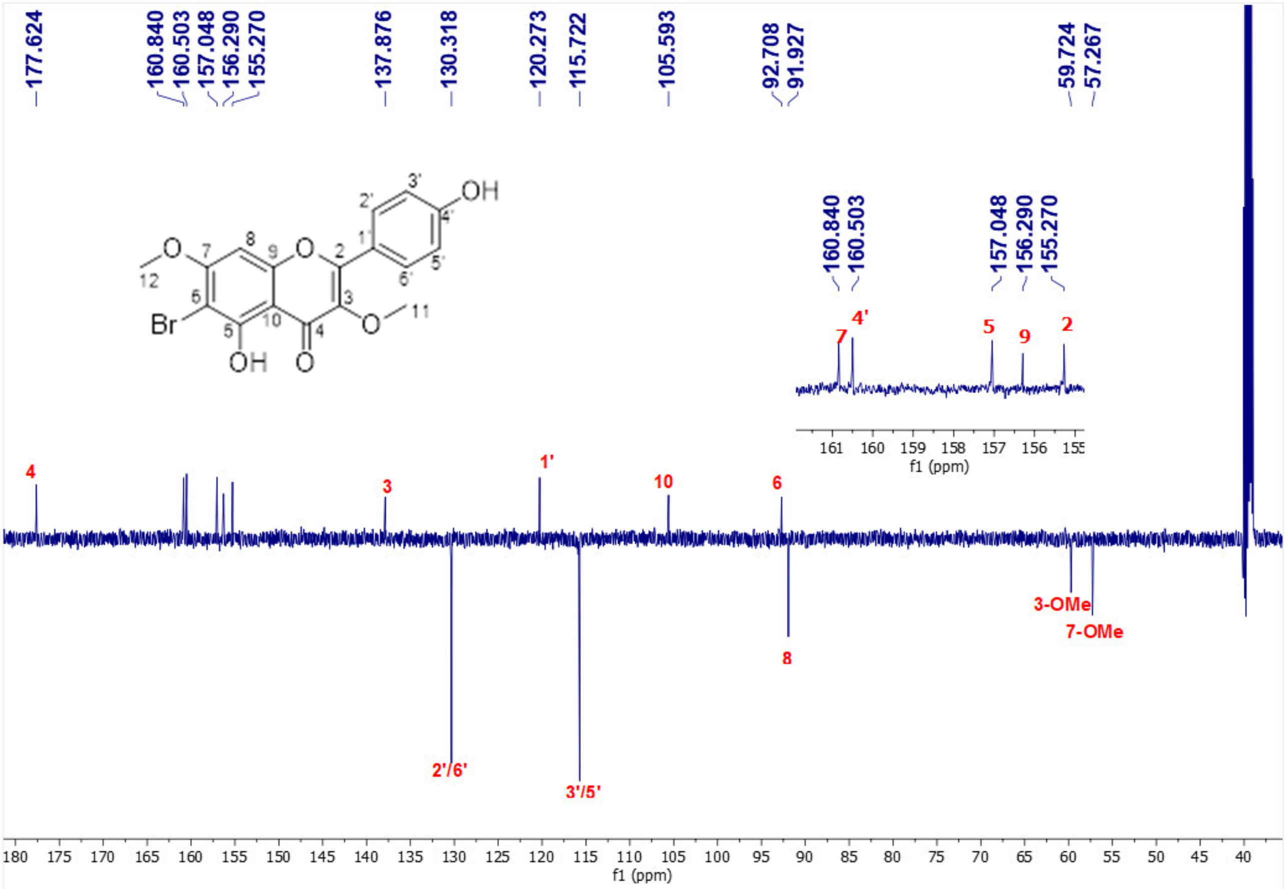

Figure S10. The ^{13}C NMR spectrum of **10** in DMSO--*d*6

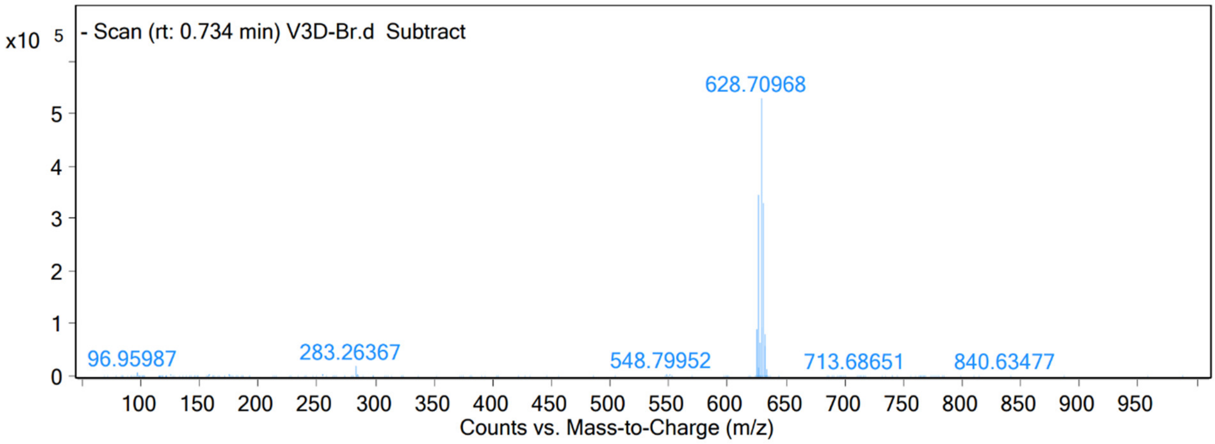

Figure S11. The HRESIMS spectrum of **11**

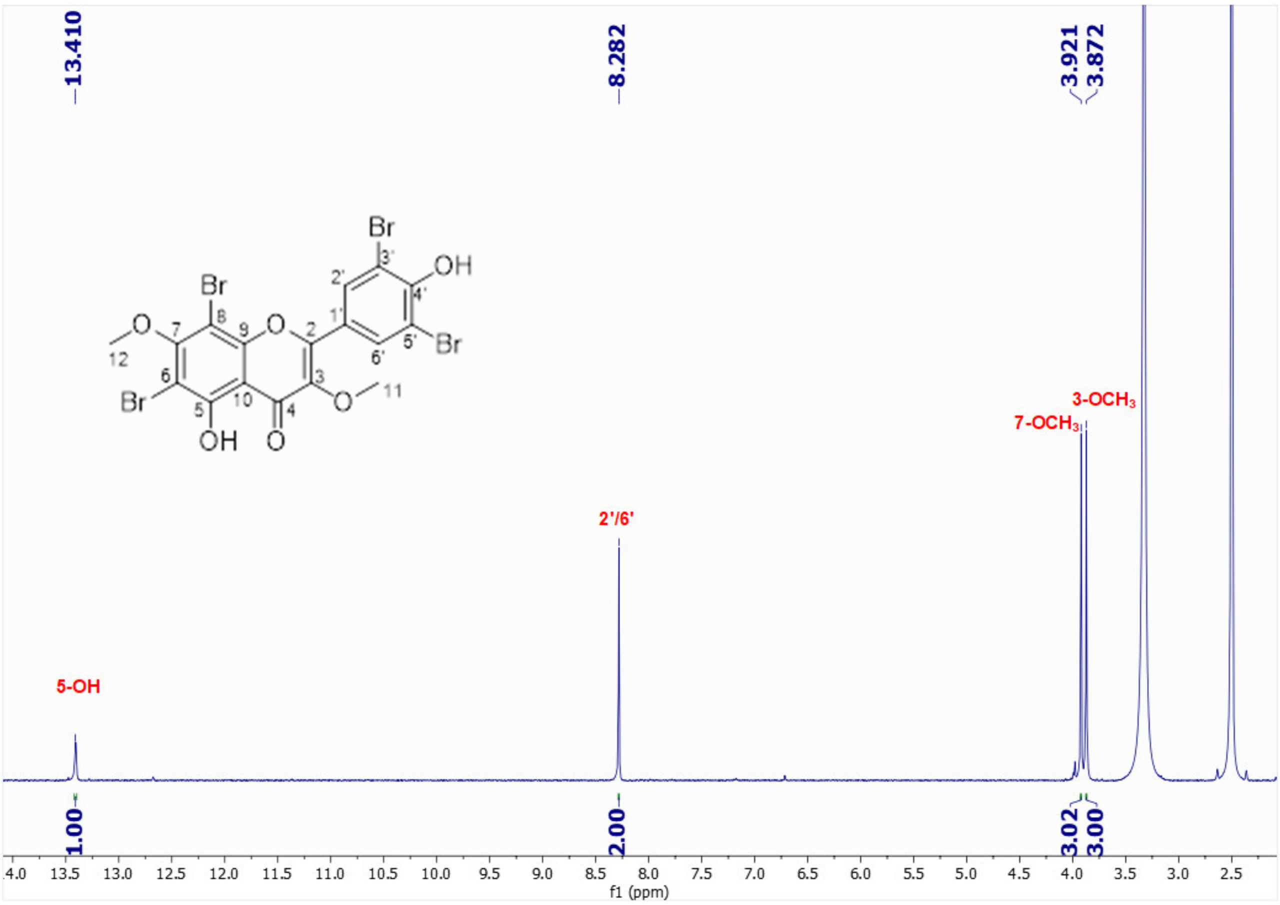

Figure S12. The ^{1}H NMR spectrum of **11** in DMSO--*d*6

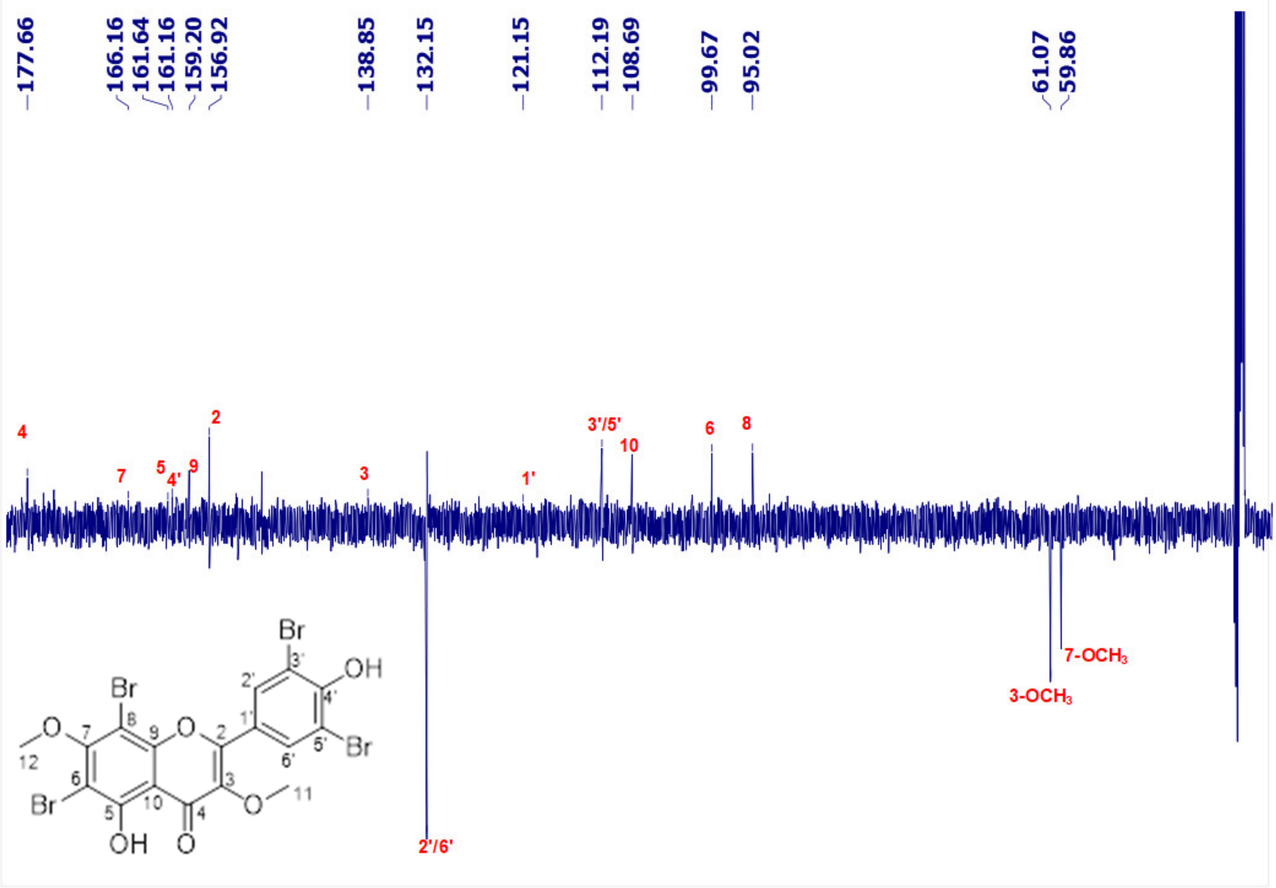

Figure S13. The ^{13}C NMR spectrum of **11** in DMSO--*d*6

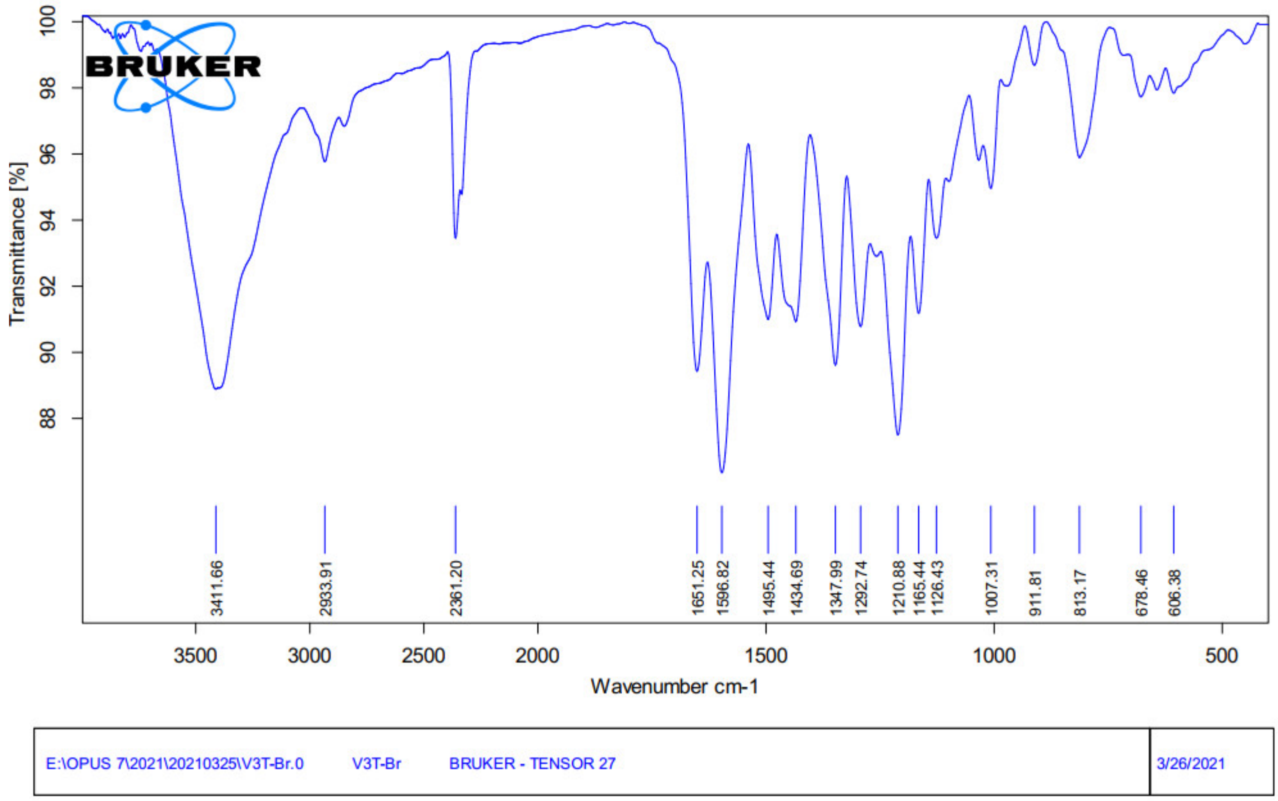

Figure S14. The IR spectrum of **8**

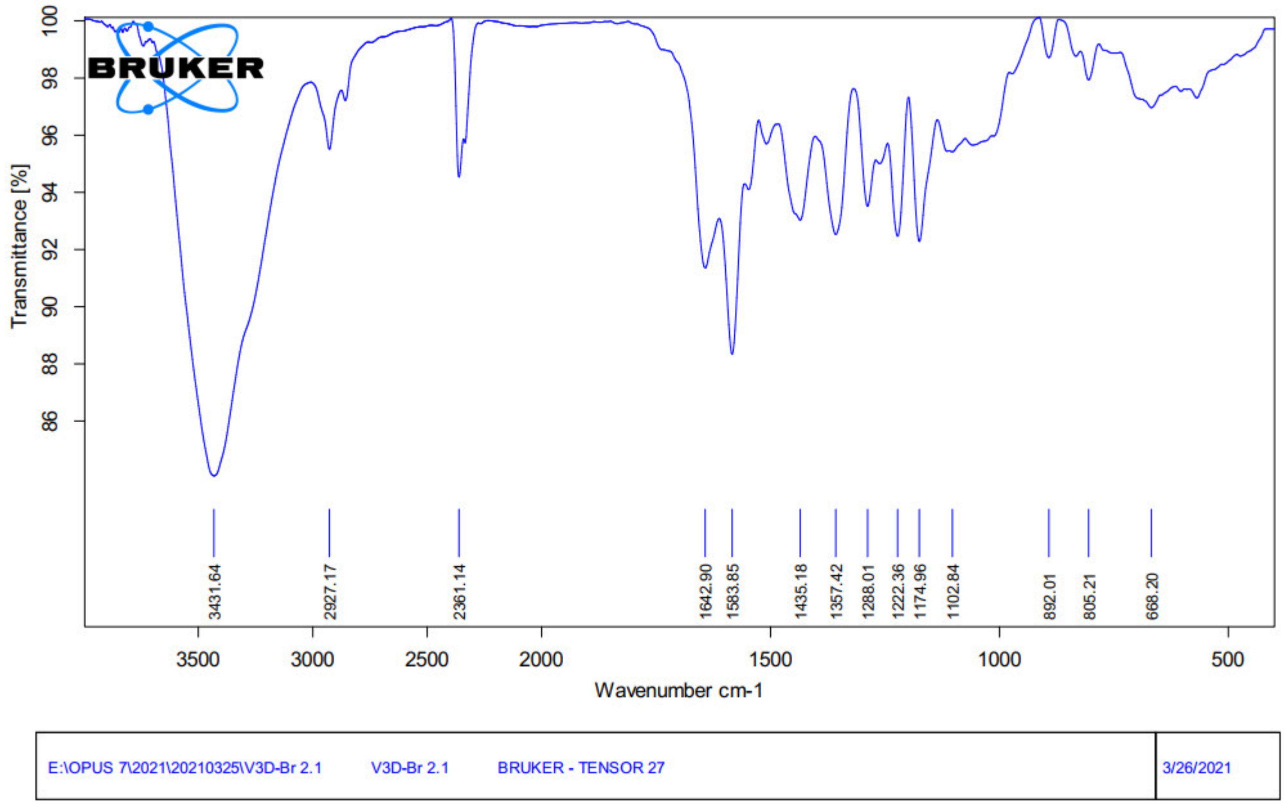

Figure S15. The IR spectrum of **9**

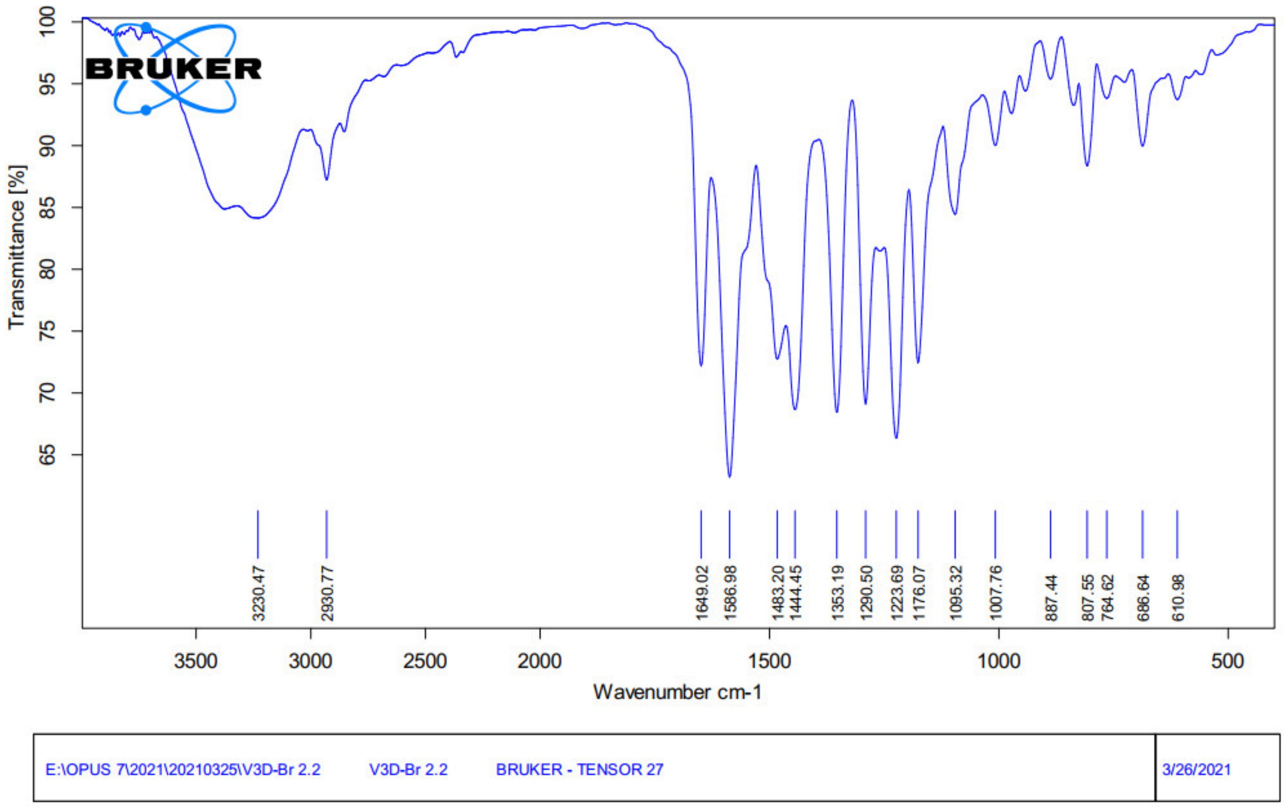

Figure S16. The IR spectrum of **10**

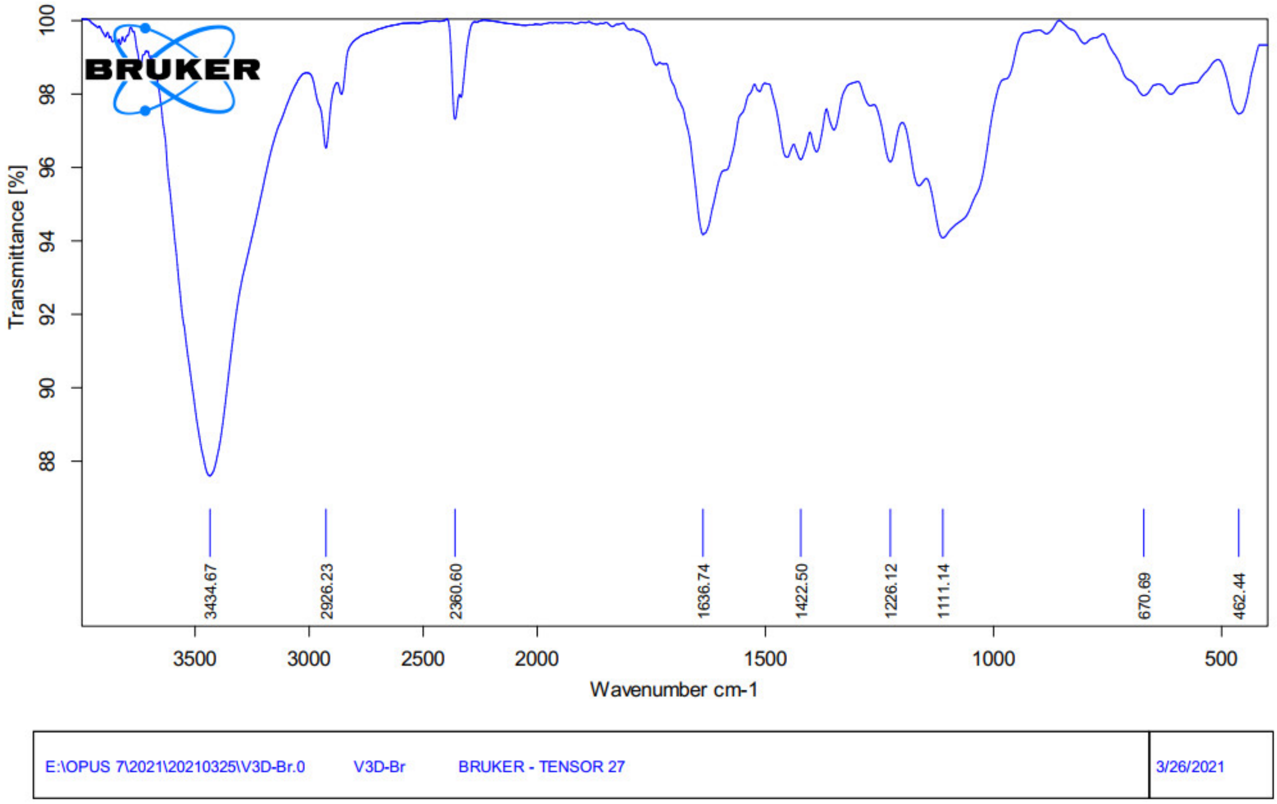

Figure 17. The IR spectrum of **11**

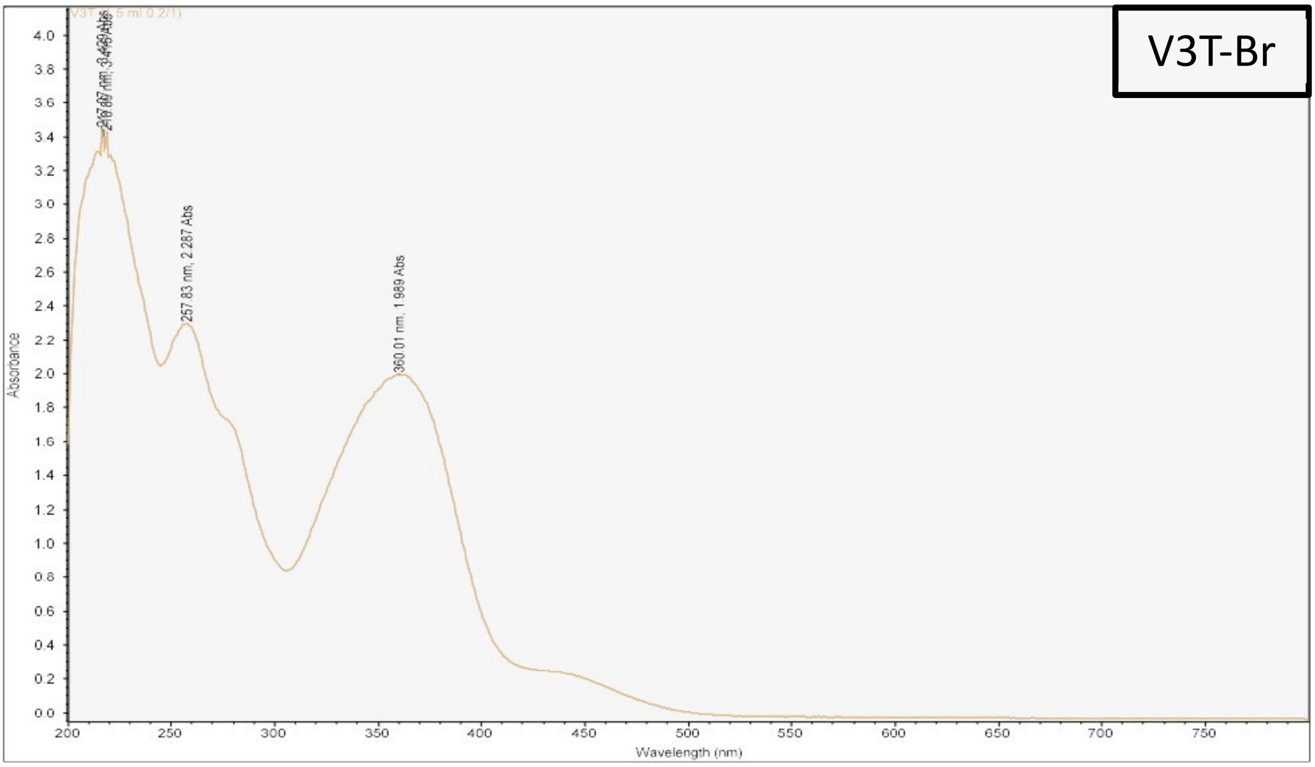

nm	Abs
217.070	3.429
218.894	3.415
257.828	2.287
360.014	1.989

Figure S18. The UV spectrum of **8**

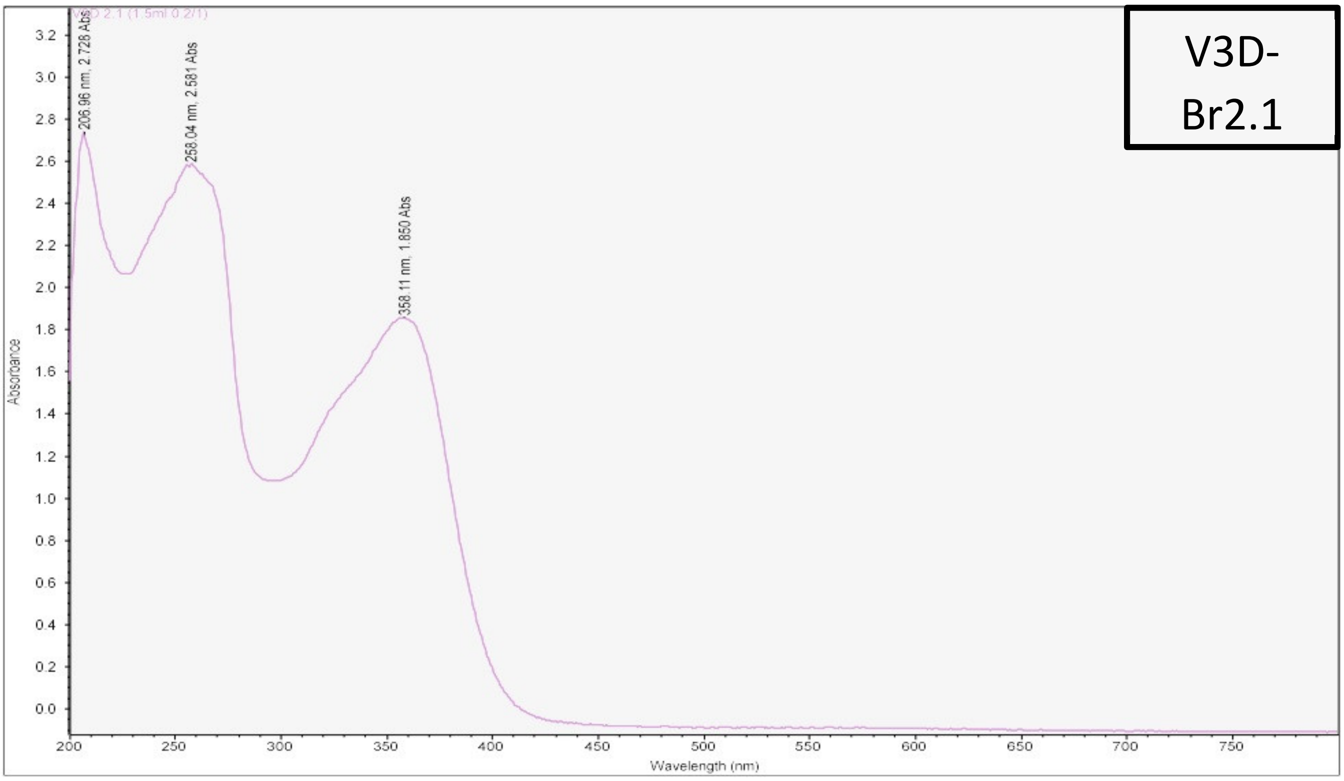

nm	Abs
206.956	2.728
258.037	2.581
358.108	1.850

Figure S19. The UV spectrum of **9**

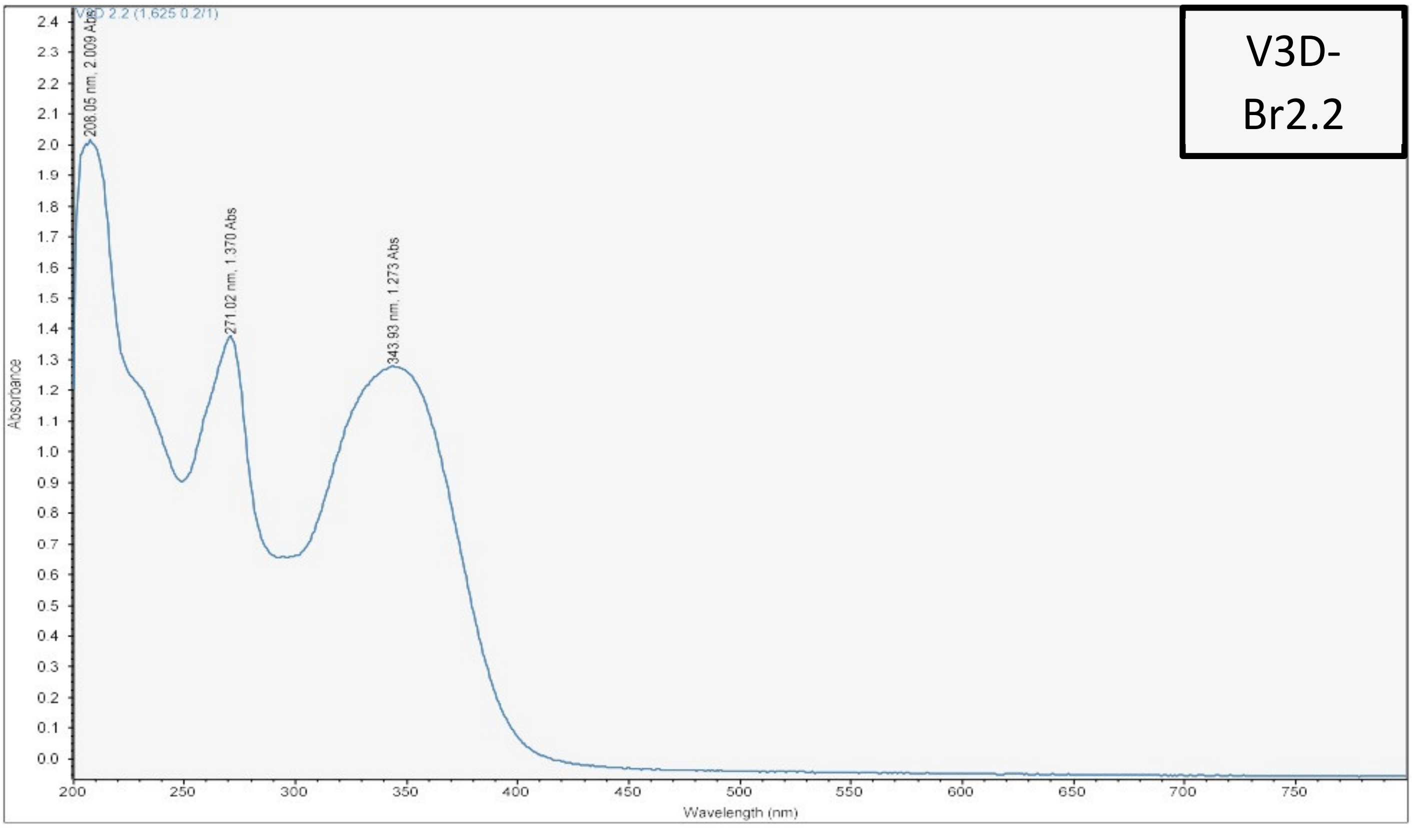

nm	Abs
208.052	2.009
271.020	1.370
343.929	1.273

Figure S20. The UV spectrum of **10**

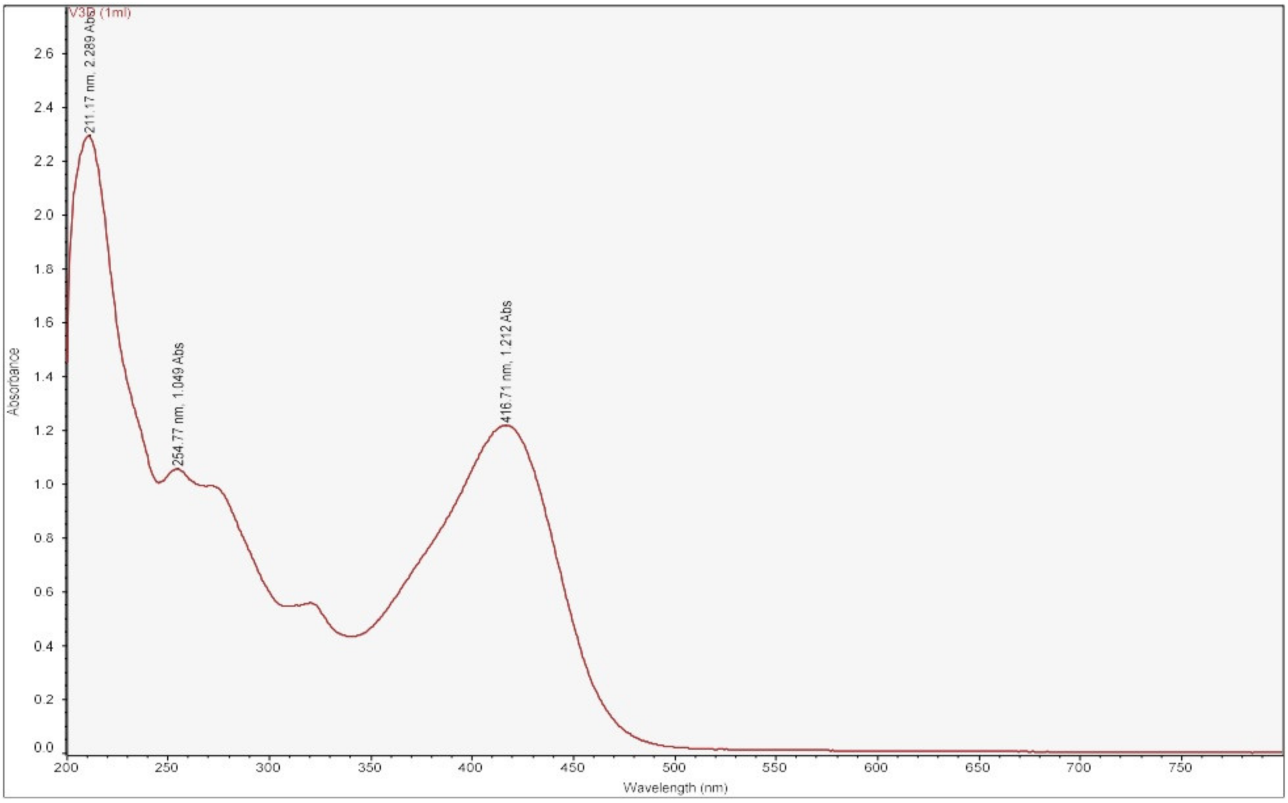

nm	Abs
211.168	2.289
416.706	1.212
254.770	1.049

Figure S21. The UV spectrum of **11**

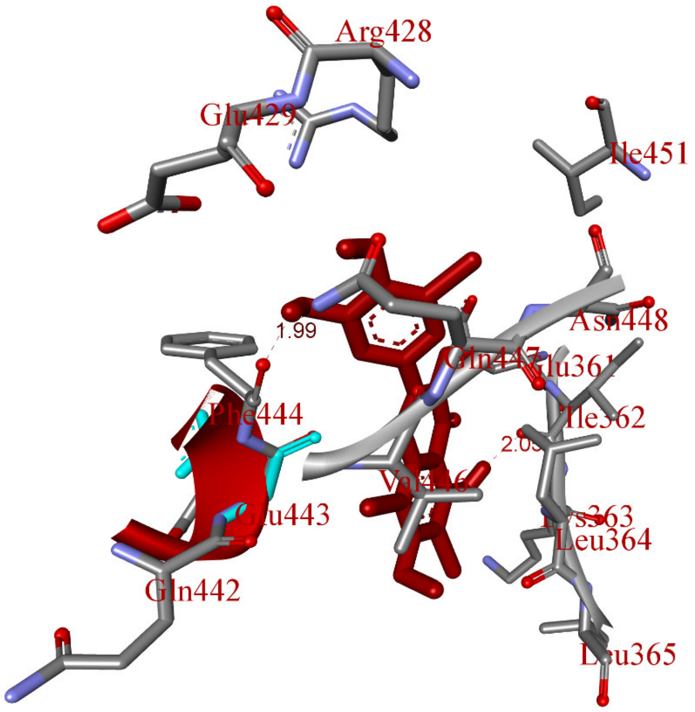

Figure S22. The residual amino acids formed around the most stable conformation ligand, **8** .

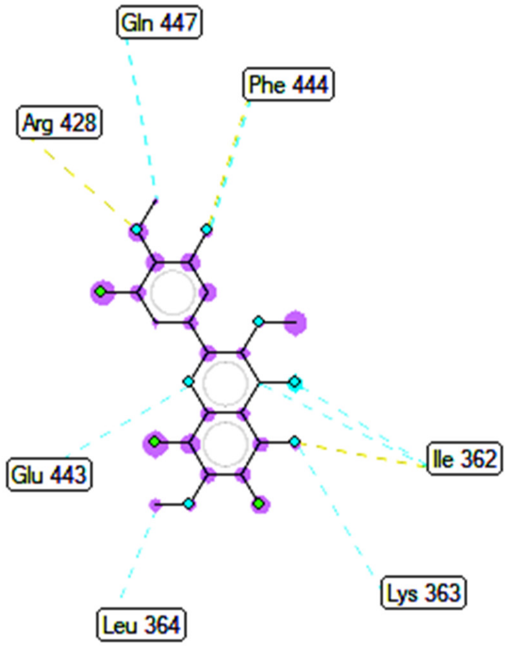

Figure S23. The ligand map indicated the significant interaction such as hydrogen bond, steric, and overlap between **8** and 4J5T.

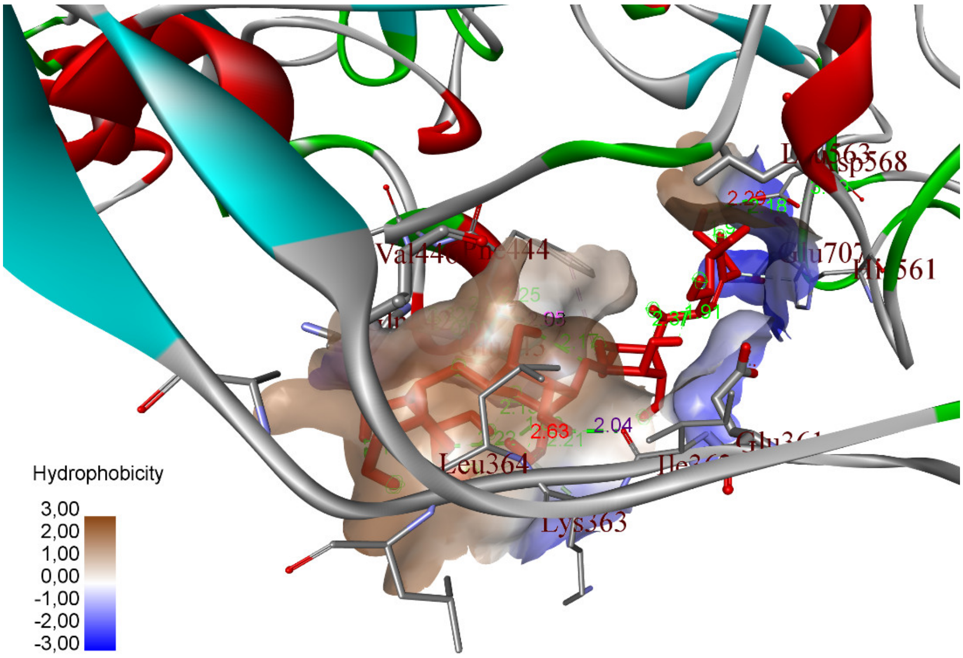

Figure S24. The active site atoms on the most stable pose of **Acarbose** was linked to pocket of receptor, 4J5T.

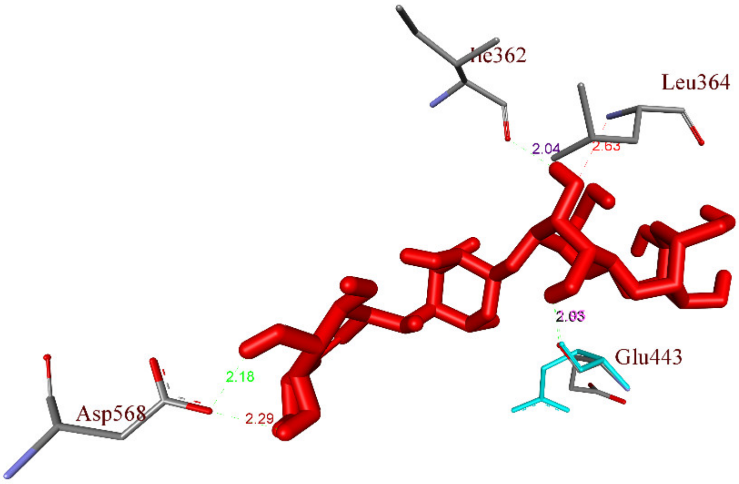

Figure S25. The hydrogen bonds was formed from residual amino acids of receptor to a docking pose of **Acarbose**.

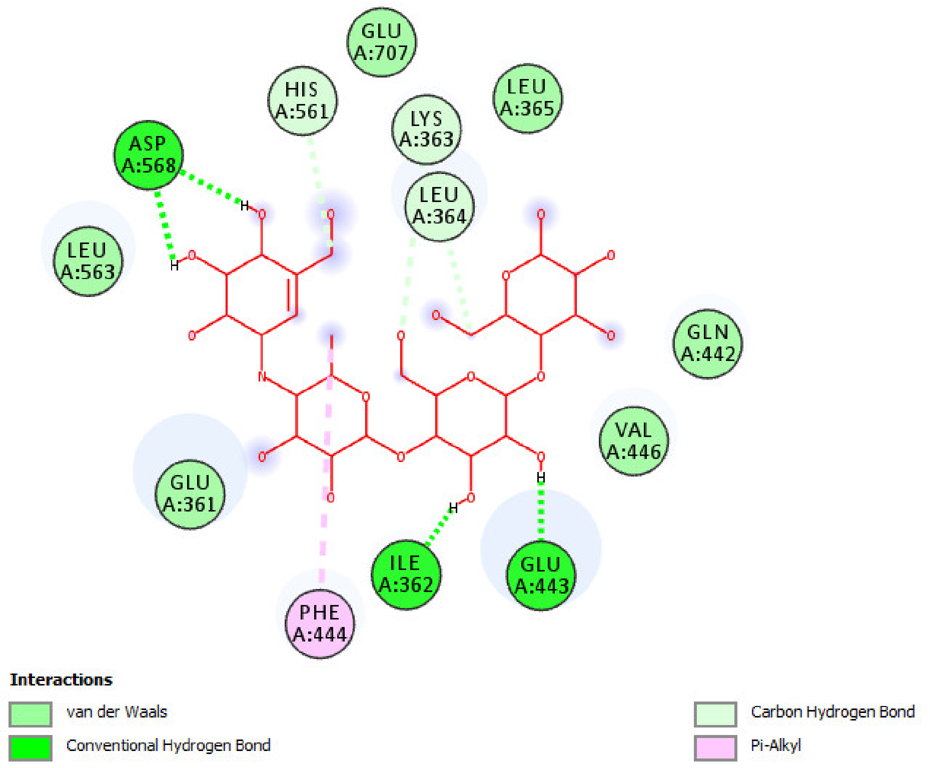

Figure S26. The 2D diagram was indicated the significant interactions between conformation ligand and enzyme, 4J5T.

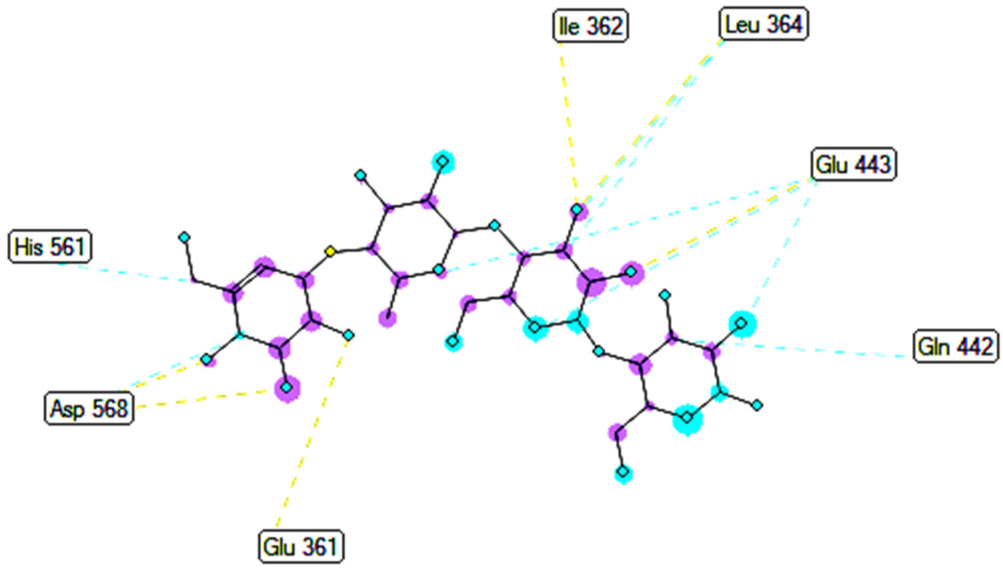

Figure S27. The ligand map exposed the significant interactions as hydrogen bond, steric, and overlap of acarbose and receptor.

General procedure for in *silico* molecular docking model

The high active compounds *in vitro*, **11**, **8**, and Acarbose (drug), which exposed the potential inhibitions against enzyme α-glucosidase were continuously performed *in silico* the molecular docking model. The crystal structures of a target, a target enzyme code **4J5T: PDB** were downloaded from the Protein data. The parameters were set up such as the grid Point Spacing of 0.586 Angstroms, the number of User-specified Grid Points of (120 x-points, 110 y-points, 118 z-points), coordinates of central grid point of maps of (-17.921, -18.441, 10.279). The parameters of input and output in docking processing were selected the genetic algorithm and Lamarckian methods, respectively. The maximum negative value of free energy of binding was selected corresponding to the most stable conformation after 2500000 energy evaluations and run 200 times, 200 models for dockings. The calculation processing, which performed based on autodock package and run form DOS commands such as **autogrid4.exe -p dock.gpf -l dock.dlg & and autodock4.exe -p dock.dpf -l dock.dlg &**, respectively. The models of significant interactions between the most conformation ligand and receptor/target protein were built by Discovery Studio 2019 Client and Molegro Molecular Viewer packages.

Results of docking calculation of high activity conformation ligand, 11, 8, and Acarbose indicated in Table 1.

Table S5. The significant results *in* silico molecular docking model of conformation ligands, docking poses, have been docked to receptor or target of crystal structure of enzyme alpha-Glucosidase and presented in the concepts of total amount of energy, free energy of binding, the inhibitor constant of fifty percentage (K_i), the number of hydrogen bond, property, and the length bond of a hydrogen bond in unit of Å.

Entry	The total amount of energy[a]	Free Energy of Binding[b]	K_i[d]	The number of hydrogen bonds[e]	The property and bond length[f]
**11**	**120,85**	**-9.45**	**0.118**	**4**	A:ARG428:N - **:11**:O (2.91)<br>A:ASN448:N - : **11** :BR22 (3.14 Å)<br>: **11** :H - A:ILE362:O (1.83)<br>: **11** :H - A:GLN447:O(2.10 Å)
**8**	**131,39**	**-8.83**	**0.337**	**2**	: **8** :H - A:ILE362:O (2.05 Å)<br>: **8** :H - A:PHE444:O (1.98 Å)
**Acarbose**	163.37	-4.55	461	**6**	A: LEU 364: N -: **Acarbose**:O (2.63)<br>: **Acarbose**: H - A:ASP568:O (2.29)<br>: **Acarbose**: H - A:ASP568:O (2.18)<br>: **Acarbose**: H - A: GLU443:O (2.03)<br>: **Acarbose**: H - A: GLU443:O (1.95)

Entry	The total amount of energy[a]	Free Energy of Binding[b]	K_i[d]	The number of hydrogen bonds[e]	The property and bond length[f]
					: **Acarbose**: H - A: ILE362:O (2.04)

[a]. The values in unit of kcal.mol^{-1} were reported by the Avogadro software after optimal processing via force field method- MMFF94. [b]. They were calculated by AutoDockTools–1.5.6rc3 package and reported in unit of kcal.mol^{-1}. [c]. The inhibition constants, K_i, μM were conducted by AutoDockTools–1.5.6rc3 package. [d], [e]. They were built based on Discovery Studio (DSC) software.

Table S6. α-Glucosidase inhibitory activity of fractions

Fractions	IC_{50} (μg/mL)
**Methanol crude**	4.0
**H**	>100
**EA**	4.6
**HEA**	0.85
**M**	>100
**P1**	>50
**P2**	11
**P3-P8**	>50
**P9**	34
